# Identification of QTL controlling volatile terpene contents in tea plant (*Camellia sinensis*) using a high-aroma ‘Huangdan’ x ‘Jinxuan’ F_1_ population

**DOI:** 10.3389/fpls.2023.1130582

**Published:** 2023-03-29

**Authors:** Si Chen, Xuanye Li, Yujie Liu, Jiedan Chen, Jianqiang Ma, Liang Chen

**Affiliations:** Key Laboratory of Biology, Genetics and Breeding of Special Economic Animals and Plants, Ministry of Agriculture and Rural Affairs, Tea Research Institute of the Chinese Academy of Agricultural Sciences, Hangzhou, China

**Keywords:** tea plant, linkage analysis, quantitative trait loci, volatile terpene, RNA-seq

## Abstract

Aroma is an important factor affecting the character and quality of tea. The improvement of aroma trait is a crucial research direction of tea plant breeding. Volatile terpenes, as the major contributors to the floral odors of tea products, also play critical roles in the defense responses of plants to multiple stresses. However, previous studies have largely focused on the aroma formation during the manufacture of tea or the comparison of raw tea samples. The mechanisms causing different aroma profiles between tea cultivars have remained underexplored. In the current study, a high-density genetic linkage map of tea plant was constructed based on an F_1_ population of ‘Huangdan’ × ‘Jinxuan’ using genotyping by sequencing. This linkage map covered 1754.57 cM and contained 15 linkage groups with a low inter-marker distance of 0.47 cM. A total of 42 QTLs associated with eight monoterpene contents and 12 QTLs associated with four sesquiterpenes contents were identified with the average PVE of 12.6% and 11.7% respectively. Furthermore, six candidate genes related to volatile terpene contents were found in QTL cluster on chromosome 5 by RNA-seq analysis. This work will enrich our understanding of the molecular mechanism of volatile terpene biosynthesis and provide a theoretical basis for tea plant breeding programs for aroma quality improvement.

## 1 Introduction

Tea is one of the most popular nonalcoholic beverages in the world, made by tender shoots of tea plant. Aroma is a critical factor influencing the quality of tea products, affecting consumer preference (Yang et al., 2013). In addition to the manufacturing process and environmental factors, tea cultivars are one of the key factors affecting the aroma of tea products (Zheng et al., 2016). As early as 1981, it was reported that the concept of terpene index (TI) was proposed by studying the aroma characteristics and systematic variation among tea cultivars, pointing out that the content of monoterpenes, such as linalool and geraniol, were species-specific (Takeo, 1981). Another report found that the varietal differences of aromatic profile remain significant for tea cultivars with very close genetic background (Lin et al., 2013). A recent study on ten tea cultivars with different leaf areas demonstrated a positive correlation between linalool content and leaf area (Zeng et al., 2021). Therefore, selecting high-aroma cultivars is one of the most interesting targets for tea plant breeding. In comparison to conventional breeding methods, marker assisted selection (MAS) through the discovery of favorable alleles and application of suitable molecular markers can greatly shorten the breeding process to accelerate the traditional tea plant breeding. However, previous studies have largely focused on the aroma formation during the manufacture of tea or the comparison of raw tea samples (Feng et al., 2019; Shi et al., 2019; Yu et al., 2021). The mechanisms causing different aroma profiles between tea cultivars have remained underexplored. The quantitative trait loci (QTL) mapping and candidate gene identification of aroma traits in tea plant have not yet been reported, which has become a technical bottleneck for efficient genetic breeding of high-aroma tea cultivars.

Volatile monoterpenes and sesquiterpenes are important components of tea aroma, often with strong floral, sweet, and woody scents. For example, geraniol has a sweet, oral aroma similar to that of roses, while linalool has a lily-like aroma, which are particularly important for the aroma quality of green, black, and oolong teas (Wang et al., 2017; Guo et al., 2022; Shi et al., 2022). Apart from being a major contributor to the floral odors of plant products, terpenoids have been shown to have a variety of biological activities (Singh and Sharma, 2014). Terpene volatiles in tea plant such as *α*-farnesene and ocimene, play important roles in plant-to-plant communication (Zeng et al., 2017). Sesquiterpene (*E*)-nerolidol was found to be a volatile signal involved in response to abiotic and biotic stresses (Chen et al., 2020a; Zhao et al., 2020). Although more and more studies in recent years have focused on the metabolic mechanism of volatile terpenes, the research on the synthesis and regulation of volatile terpenes in tea plant still has to be improved compared with model plants (Mei et al., 2017; Liu et al., 2018; Xu et al., 2018b; Liu et al., 2020).

The tea plant is basically self-incompatible with high heterozygosity and long growth cycle (Zhang et al., 2016). Hence it is difficult to generate a population fitting for QTL mapping, such as recombinant inbred lines (RIL) and chromosome segment substitution lines. Although several genetic maps were reported in tea plant, most of them chose hybrid parents cultivars based on the content of nonvolatile metabolites, such as flavonoid, caffeine, and amino acid (Ma et al., 2018; Xu et al., 2018a; Huang et al., 2022b). Therefore, a more suitable population is needed for the QTL mapping of aroma compounds. In this study, we initially constructed an F_1_ population from a controlled cross between the high-aroma tea cultivar ‘Huangdan’ ('HD') and ‘Jinxuan’ ('JX'). As a reference standard for breeding oolong tea cultivars in China, 'HD' is well known for its high levels of aroma. It originated from Fujian Province and has been used to breed a series of excellent hybrid offspring (Wang et al., 2022b). The other parent 'JX' is an oolong tea that originated from Taiwan Province with a unique fragrance. To elucidate the molecular mechanism of aroma differences among tea cultivars and the key genes controlling the accumulation of volatile terpenes, we constructed a high-density SNP linkage map using 148 individuals of F_1_ population. Headspace solid-phase microextraction (HS-SPME) combined with gas chromatography-time-of-flight mass spectrometry (GC-TOF MS) was used to analyze the aroma components. The major differential volatile terpene compounds and their genetic variation were investigated in the parents and their F_1_ progeny. Further QLT mapping and comparative transcriptomics analyses were performed to screen the candidate genes associated with terpenoid contents. This work will enrich the theoretical basis of the molecular mechanism of volatile terpene biosynthesis and breeding of high-aroma tea cultivars.

## 2 Materials and methods

### 2.1 Plant materials

The mapping population composed of 148 F_1_ individuals was derived from a controlled cross between ‘Huangdan’ ('HD') and ‘Jinxuan’ ('JX'). The parents and all F_1_ individuals were planted in the Shengzhou Experimental Station of Tea Research Institute of the Chinese Academy of Agricultural Sciences. The healthy ‘two and a bud’ (apical bud with two adjacent young leaves) tea samples were collected from each tea plant for DNA extraction, RNA extraction, and aroma analysis in the spring of 2020 and 2021.

### 2.2 SNP identification by genotyping by sequencing and linkage map construction

Approximately 0.1 g of each tea sample was ground in liquid nitrogen for genomic DNA isolation using an improved CTAB method (Dellaporta et al., 1983). The DNAs of 'HD', 'JX', and 148 F_1_ individuals were digested by *Nla*III and *Msp*I restriction enzymes, and the genotyping by sequencing (GBS) libraries were constructed. After purification, the GBS libraries was sequencing using Illumina Novaseq 6000 platform (Novogene, Beijing, China). Clean data were obtained by removing low quality reads and then aligned to the genome of the parent 'HD' *via* BWA and SAMTOOLS software. The genome sequences and gene annotation files were downloaded in the BIG Data Center (https://bigd.big.ac.cn/) under project number PRJCA003382 (Wang et al., 2021). SNP calling was performed for all samples using the GATK software. The SNPs were screened by filtering out false-positive SNPs and those showed distorted segregation (*P* < 0.001, χ2 test). Using the high-quality SNP markers, the genetic map was constructed using JoinMap (Ver. 4.1, Kyazma, Wageningen, Netherlands) with the maximum-likelihood method.

### 2.3 GC-TOF MS analysis

Extraction of volatiles from tea samples was achieved by HS-SPME as previously described with some modifications (Chen et al., 2021). Briefly, 0.6 g of samples were ground to fine powders with the precooled miller after lyophilization, placed into a 20 mL sealed headspace vial and incubated at 80°C for 2 min before extraction. A 1 cm, 65 μm 50/30 μm divinylbenzene/carboxen/polydimethylsiloxane (DVB/CAR/PDMS) SPME fiber (Supelco, Bellefonte, PA, USA) was used to collect volatiles for 60 min at 80°C. All volatile compounds absorbed onto the SPME fiber were desorbed at 250°C for 5 min in splitless mode and analyzed using an Agilent 7890B gas chromatograph (Agilent Co., Santa Clara, CA, USA) coupled with a Pegasus 4D time-of-flight mass spectrometer (LECO Co., Saint Joseph, MI, USA) (GC-TOF MS). A Restek Rxi^®^-5Sil MS capillary column (30 m×0.25 mm×0.25 µm film thickness) was employed with high purity helium as the carrier gas at a flow rate of 3 mL/min. The injection and transfer line temperature were controlled at 250°C. During the analysis, the oven temperature program started at 50°C and maintained for 2 min, increasing to 265°C at a rate of 4°C/min and maintaining for 5 min. The mass spectra ion source temperature and electron energy were set to 220 °C and -70 eV, respectively. The detector voltage was 1,550 V. The scan range was 33-500 atomic mass unit (AMU) with 10 spectra/s acquisition rate. Three biological repetitions were performed for volatile extraction. Quality control (QC) samples were prepared by mixing 1.0 g of every sample to become a combined sample. QC samples were injected throughout the analytical runs (every five samples) to check the instrument performance.

Raw data acquired by GC-TOF MS were processed by ChromaTOF software (Ver. 4.51.6, LECO, St. Joseph, MI, USA) with default settings. Aroma compounds were identified or tentatively identified by comparing their retention time, retention indices (RI) and mass spectra with those of authentic standards or against the NIST library (http://webbook.nist.gov/). Quantitative measurements of volatiles were based on the mean area of selected characteristic ions after normalization. The orthogonal partial least squares-discriminant analysis (OPLS-DA) was performed to identify differential volatile metabolites using Simca-P (Ver. 14.1, Umetrics AB, Umeå, Sweden) after Pareto scaling. Violin plots of the distribution of the main aroma compounds in F_1_ population were made using Python program seaborn.

### 2.4 QTL mapping

QTL mapping was performed using MapQTL (Ver. 6.0, Kyazma, Wageningen, Netherlands) with the interval mapping (IM) method. A LOD threshold of 3.0 was set to identify significant QTL. Ranges above the LOD of 3.0 were identified as QTL intervals (Wang et al., 2019b). The QTL detected across years for each trait was assigned to be the same stable QTL if confidence intervals overlapped. QTL positions were drawn using MapChart (Ver. 2.32, Wageningen, Netherlands).

### 2.5 Gene expression analysis using RNA-seq and qRT-PCR

Approximately 0.1 g of tea samples (three biological replicates per genotype) were used for RNA extraction. Total RNAs were extracted with the RNAprep Pure Plant Kit (Tiangen, Beijing, China) following the manufacturer’s procedures. Sequencing libraries were generated and paired-end 150 bp sequencing was performed on an Illumina Novaseq 6000 platform using a customer sequencing service (Novogene, Beijing, China). Clean reads were obtained by removing reads containing adapter, reads containing ploy-N and low quality reads from raw data. The index of the reference genome was built and paired-end clean reads were aligned to the reference genome (Wang et al., 2021) with the Hisat2 (Ver. 2.0.5). Read numbers mapped to each gene of the reference genome were counted using featureCounts (Ver. 1.5.0). Fragments per kilobase of exon model per million mapped reads (FPKM) were used to quantify transcript abundance. The DESeq2 R package (Ver. 1.20.0) was applied to identify differentially expressed genes (DEGs) with Padj < 0.05 and an absolute value of fold change > 1.5. The heatmap was generated with TBtools (Chen et al., 2020b) to visualize expression patterns of DEGs between samples.

Candidate gene transcript expressions were validated by quantitative real time PCR (qRT-PCR). The cDNA was reversely transcribed using PrimeScript^TM^ RT reagent Kit (Takara, Dalian, China). Gene specific primers of candidate genes were designed and given in **Table S3**. Relative gene expression was calculated by the 2^-ΔΔCT^ method using the *CsGAPDH* sequence (accession no. KA295375) as the reference gene.

## 3 Results

### 3.1 Genotyping by sequencing of the F_1_ population

A total of 139.34G clean bases were derived from sequencing assembly of 148 F_1_ individuals using the GBS approach with an average complete digestion rate was 90.24%. The Average Q20, Q30, and GC content were 95.35%, 87.96%, and 36.38% respectively, proving the high quality of the sequencing results. The 985.52 Mb clean reads of F_1_ offspring were obtained by aligning with the 'HD' reference genome with an average matching rate of 98.81%. The average read depth was 9.04×. Based on the genotyping result of two parental lines, a total of 11,462,958 single nucleotide polymorphisms (SNPs) were found. Among them, only segregation types lm × ll (2,001,025), nn × np (2,918,476), and hk × hk (6,380,260) were selected for genotyping in F_1_ individuals. The final number of available SNPs was 11,299,761 (**Figure S1**). After removing abnormal bases and deleting segregation distortion (*P* < 0.001), we obtained 13,289 effective SNPs used for linkage analysis.

### 3.2 Construction of a high-density linkage map

**Figure 1:**
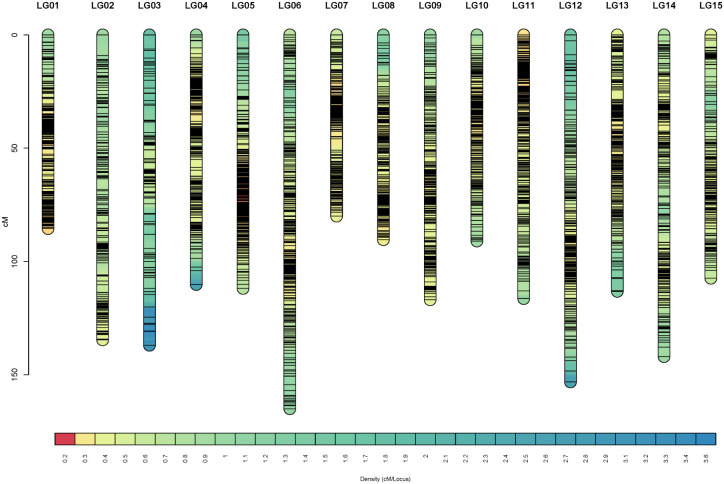
Marker distribution across the linkage map. The x-axis, y-axis, and lines in the column represent the linkage group, genetic distance, and unique marker, respectively.

As a result, 3770 SNP markers were finally mapped into 15 linkage groups (LGs), corresponding to the haploid chromosome number of tea plant (**Figure 1**). The linkage map covered a total genetic distance of 1754.57 cM, with an average marker distance of 0.47 cM. The average length of the 15 linkage groups was 116.97 cM. The longest linkage group was LG06 (165.01 cM), and the shortest was LG07 (79.99 cM). A total of 99.68% marker interval size was below 5 cM, and only 12 of them were between 5 and 10 cM (**Table 1**). The information on 3770 markers in the linkage map was presented in **Table S1**.

**Table 1:** Information and statistics of the genetic map.

Group	SNP number	Genetic distance (cM)	Average distance (cM)	max_gap (cM)
LG01	258	85.46	0.33	2.29
LG02	201	134.57	0.67	9.25
LG03	123	137.05	1.11	6.64
LG04	258	110.16	0.43	6.04
LG05	308	111.92	0.36	5.76
LG06	312	165.01	0.53	3.86
LG07	258	79.99	0.31	5.01
LG08	236	90.38	0.38	4.57
LG09	268	116.93	0.44	3.38
LG10	226	90.99	0.40	4.62
LG11	326	116.42	0.36	4.33
LG12	247	153.16	0.62	6.16
LG13	284	113.19	0.40	5.29
LG14	264	141.97	0.54	4.37
LG15	201	107.38	0.53	3.65
Total	3770	1754.57	0.47	9.25

### 3.3 Phenotypic variations of volatile terpene contents

To clarify the differences in terpene volatile contents between 'HD' and 'JX'. The aroma compound phenotypes of two parental lines were measured by HS-SPME GC-TOF MS in 2020 and 2021. The OPLS-DA model was built using two years of data respectively to select differential volatile metabolites that make a significant contribution to classification. As a result, 12 volatile terpenes showed significant differences (*P* < 0.05 and at least one year VIP > 1), including 8 monoterpenes ((*E*)-*β*-ocimene, *cis*-linalool oxide (furanoid), *trans*-linalool oxide (furanoid), linalool, *trans*-linalool oxide (pyranoid), *α*-terpineol, (*Z*)-nerol, and geraniol) and 4 sesquiterpenes (*α*-cubebene, *δ*-cadinene, calamenene, and (*E*)-nerolidol) (**Table 2**). The contents of these 12 volatile terpenes were higher in 'HD' than in 'JX' in both years. Among them, geraniol was the most significantly different volatile terpene. The VIP values were greater than 10 for both years (10.84 in 2020, 15.19 in 2021). In addition, linalool and *trans*-linalool oxide (furanoid) also showed significant differences with both VIP values and fold changes higher than 2 in these two years.

**Table 2:** Phenotypic variation of the volatile terpene contents in the ‘HD’ and ‘JX’ parents and F_1_ progeny.

Num	Compound	RT (s)	RI ^a^	RI ^b^	Year	Parents		F_1_ population		
			calculated	published		VIP	FC HD/JX	CV (%)	Kurtosis	Skewness
Monoterpenes										
1	(*E*)-*β*-Ocimene	812.87	1041.5	1043	2020	0.48	1.67	79.40	7.04	2.29
					2021	1.14	6.54	63.89	2.93	1.76
2	*cis*-Linalool oxide (furanoid)	868.67	1068.0	1070	2020	1.35	1.34	61.31	0.96	1.04
					2021	1.69	3.03	62.26	10.03	2.43
3	*trans*-Linalool oxide (furanoid)	905.54	1085.5	1086	2020	3.51	2.04	66.03	0.23	0.93
					2021	2.53	4.34	56.72	7.30	2.10
4	Linalool	933.41	1098.8	1098	2020	4.94	2.23	57.94	5.06	1.79
					2021	3.04	3.11	40.15	3.25	1.43
5	*trans*-Linalool oxide (pyranoid)	1098.65	1175.9	1173	2020	2.47	2.87	57.52	2.65	1.24
					2021	1.65	2.31	60.06	2.52	1.42
6	*α*-Terpineol	1143.72	1197.0	1197	2020	1.42	1.60	89.31	89.46	8.51
					2021	0.83	8.41	91.13	39.79	5.19
7	(*Z*)-Nerol	1203.44	1225.4	1226	2020	0.98	1.57	73.58	7.36	2.25
					2021	1.46	6.81	80.00	4.27	1.90
8	Geraniol	1264.16	1254.3	1253	2020	10.84	3.41	101.41	4.42	2.04
					2021	15.19	8.21	76.23	1.84	1.53
Sesquiterpenes										
9	*α*-Cubebene	1459.65	1350.2	1351	2020	1.53	4.36	85.36	23.31	3.83
					2021	0.62	14.94	42.47	2.45	1.35
10	*δ*-Cadinene	1785.80	1522.0	1523	2020	2.13	3.44	67.05	23.46	3.77
					2021	0.62	28.77	62.67	1.89	1.43
11	Calamenene	1792.08	1525.5	1525	2020	1.32	4.33	58.04	12.14	2.65
					2021	0.69	20.82	53.22	2.13	1.28
12	(*E*)-Nerolidol	1856.70	1561.8	1560	2020	1.19	5.20	94.11	17.49	3.82
					2021	1.11	57.51	65.04	3.59	1.68

^a^Retention indices were calculated from a mixture of C-8 to C-20.

^b^Published retention indices on DB-5 column reported on http://webbook.nist.gov/.

**Figure 2:**
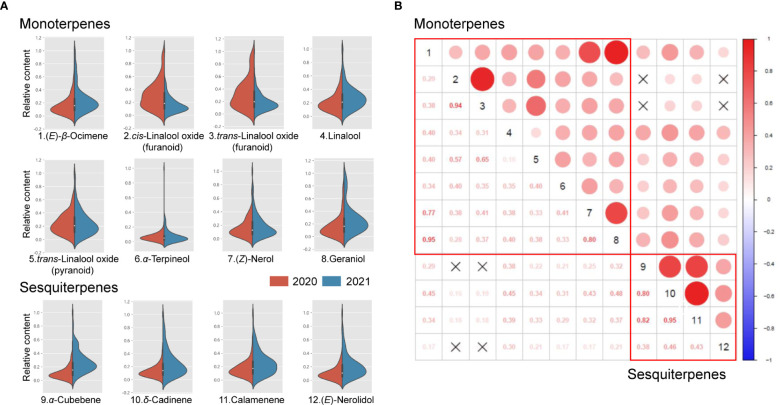
Violin-plot distribution and correlation analysis of volatile terpenes in the ‘HD’ × ‘JX’ F_1_ population. Violin-plot distribution of eight monoterpenes and four sesquiterpenes **(A)**. Pearson’s correlations among 12 volatile terpenes **(B)**. Crossed symbols indicate *P* > 0.05.

As a result, these 12 aroma compounds mentioned above were chosen to be analyzed for the phenotypic variations in the F_1_ population of 2020 and 2021. The coefficient of variation (CV), kurtosis, and skewness were calculated for each volatile terpene (**Table 2**). Significant variations of aroma compounds were found in the F_1_ population (Minimum CV > 40%). The frequency distribution of aroma compounds in the F_1_ population was illustrated in **Figure 2A**. Most aroma compounds exhibited a near-normal distribution in the F_1_ population suggested that these aroma compounds were quantitative traits. The correlations between different aroma compounds were carried out and depicted in **Figure 2B**. It was noteworthy that a significant positive correlation observed in monoterpenes and sesquiterpenes respectively, indicating that to be related to their synthetic pathways. A strong positive correlation were found between *cis*-linalool oxide (furanoid) and *trans*-linalool oxide (furanoid), as well as between (*E*)-*β*-ocimene, (*Z*)-nerol and geraniol (*r* > 0.77). In addition, *α*-cubebene, *δ*-cadinene, and calamenene also showed a strong positive correlation (*r* > 0.80).

### 3.4 QTL mapping of volatile terpene contents in the F_1_ population

**Figure 3:**
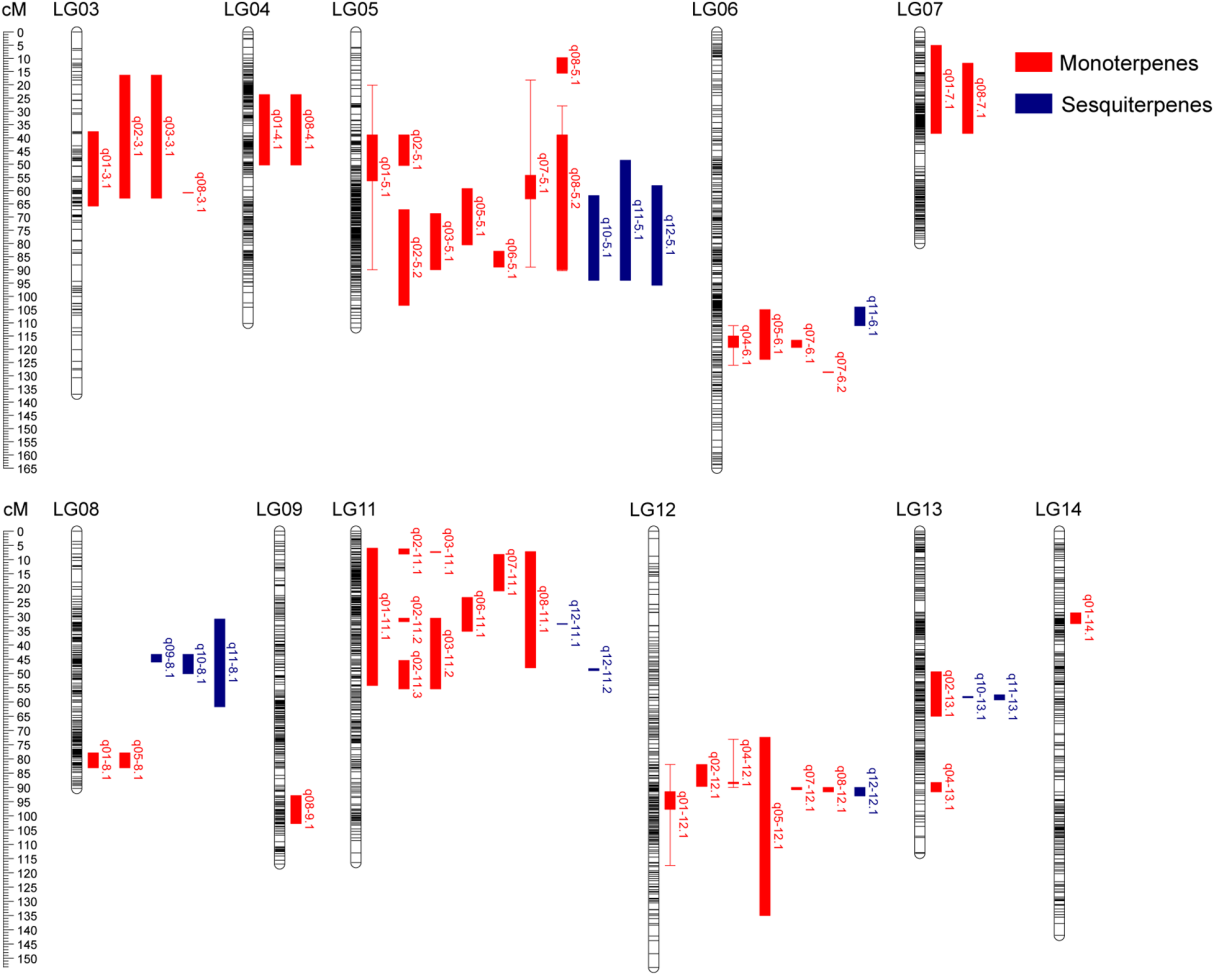
Distribution of QTL associated with volatile terpene contents identified in the ‘HD’ × ‘JX’ F_1_ population. QTL intervals are shown on the right of linkage groups, represented by bars of different color (monoterpenes, red; sesquiterpenes, blue).

We performed QTL analysis of volatile terpene contents with a phenotypic dataset from the F_1_ populations based on the linkage maps constructed in this study. A total of 54 QTLs were screened for 12 volatile terpenes. These QTLs were distributed on 11 linkage groups, including LG03-09 and LG11-13 (**Figure 3**), with a LOD score ranging from 3.08 to 6.57 (**Table 3**). The number of QTL per trait ranged from one to eight. There are eight QTLs associated with (*E*)-*β*-ocimene, *cis*-linalool oxide (furanoid) and geraniol respectively, while only one QTL controlling *α*-cubebene was identified. The proportion of phenotype variance explained (PVE) by each QTL ranged from 9.10% to 18.50%, and 47 loci had a PVE >10%. Six stable QTLs were validated in 2020 and 2021, including two for (*E*)-*β*-ocimene, two for linalool, one for (*Z*)-nerol, and one for geraniol.

**Table 3:** Overview of QTLs associated with volatile terpene contents detected in the ‘HD’ × ‘JX’ F_1_ population.

Num	Trait	QTL	LG	Year	Peak (cM)	Nearest marker	LOD	PVE%	Left marker	Right marker	Interval (cM)
1	(*E*)-*β*-Ocimene	*q01-3.1*	03	2020	60.917	np251	6.57	18.5	lm192	np190	37.709-65.818
		*q01-4.1*	04	2020	32.169	np1588	4.39	12.8	np1491	np1705	23.705-50.302
		*q01-5.1*	05	2020	56.539	np2217	4.90	14.1	np2288	np2002	38.893-89.868
				2021	28.274	np2213	5.87	16.7	np2242	np2192	20.055-56.291
		*q01-7.1*	07	2020	29.356	np3234	6.16	17.4	np3325	np3260	5.068-38.372
		*q01-8.1*	08	2021	82.048	np3705	3.75	11.0	np3615	np3706	77.885-83.074
		*q01-11.1*	11	2020	18.653	np4656	4.28	12.5	np4616	np4833	5.975-54.184
		*q01-12.1*	12	2020	90.018	np5128	4.45	12.9	hk1769	lm2506	81.968-97.698
				2021	91.578	lm2505	3.56	10.5	np5133	hk1837	91.477-117.362
		*q01-14.1*	14	2021	32.453	np5640	3.25	9.6	np5647	np5640	28.680-32.453
2	*cis*-Linalool oxide (furanoid)	*q02-3.1*	03	2020	50.794	np324	4.11	12.0	lm232	np227	16.333-62.929
		*q02-5.1*	05	2020	50.478	hk646	3.72	10.9	np2288	hk646	38.893-50.478
		*q02-5.2*	05	2020	79.254	lm982	4.75	13.7	np2143	lm942	67.172-103.417
		*q02-11.1*	11	2020	7.489	np4706	4.03	11.8	np4622	hk1544	6.221-8.072
		*q02-11.2*	11	2020	31.419	np4766	4.01	11.7	lm2222	np4767	30.625-31.796
		*q02-11.3*	11	2020	54.014	np4835	3.92	11.5	np4787	np4837	45.406-55.439
		*q02-12.1*	12	2020	89.736	np5123	3.57	10.5	hk1769	np5123	81.968-89.736
		*q02-13.1*	13	2020	49.405	np5540	3.74	11.0	np5540	np5596	49.405-65.008
3	*trans*-Linalool oxide (furanoid)	*q03-3.1*	03	2020	50.794	np324	3.72	10.9	lm232	np227	16.333-62.929
		*q03-5.1*	05	2020	79.349	np2064	3.68	10.8	np2142	np2002	68.737-89.868
		*q03-11.1*	11	2020	7.489	np4706	3.28	9.7	np4703	np4706	7.166-7.489
		*q03-11.2*	11	2020	54.014	np4835	3.89	11.4	lm2222	np4837	30.625-55.439
4	Linalool	*q04-6.1*	06	2020	119.010	np2597	3.66	10.8	np2659	hk828	115.030-119.265
				2021	124.140	np2763	3.91	11.5	np2749	np2764	111.134-126.134
		*q04-12.1*	12	2020	88.316	np5118	3.38	10.0	np5117	np5114	88.200-88.651
				2021	85.974	hk1788	4.52	13.1	np5062	np5127	73.135-90.002
		*q04-13.1*	13	2020	88.339	np5549	3.44	10.2	np5549	np5555	88.339-91.562
5	*trans*-Linalool oxide (pyranoid)	*q05-5.1*	05	2020	80.138	np2063	3.49	10.3	hk647	lm966	59.206-80.522
		*q05-6.1*	06	2020	112.828	np2675	5.56	15.9	np2662	np2759	104.999-123.767
		*q05-8.1*	08	2020	79.024	np3608	4.02	11.8	np3615	np3706	77.885-83.074
		*q05-12.1*	12	2020	78.233	hk1787	5.76	16.4	np5059	hk1864	72.357-134.952
6	*α*-Terpineol	*q06-5.1*	05	2021	82.900	lm1002	4.75	13.9	lm1002	hk606	82.900-88.853
		*q06-11.1*	11	2021	23.299	hk1585	3.70	11.0	hk1585	lm2230	23.299-35.196
7	(*Z*)-Nerol	*q07-5.1*	05	2020	55.485	np2182	3.35	10.4	np2184	np2198	54.208-63.145
				2021	68.175	np2146	5.62	16.1	np2267	hk606	18.227-88.853
		*q07-6.1*	06	2020	116.610	np2750	4.44	13.5	np2750	hk828	116.610-119.265
		*q07-6.2*	06	2021	128.750	np2625	4.09	12.0	np2624	np2625	128.542-128.750
		*q07-11.1*	11	2020	18.653	np4656	4.34	13.2	lm2194	hk1529	8.224-21.000
		*q07-12.1*	12	2020	90.018	np5128	3.82	11.7	np5128	np5104	90.018-90.847
8	Geraniol	*q08-3.1*	03	2020	60.917	np251	4.52	13.1	np245	np281	60.705-60.924
		*q08-4.1*	04	2020	42.271	np1644	3.55	10.5	np1491	np1705	23.705-50.302
		*q08-5.1*	05	2020	14.450	np2280	4.50	13.1	np2289	np2243	9.664-15.579
		*q08-5.2*	05	2020	56.539	np2217	5.15	14.8	np2288	np2002	38.893-89.868
				2021	28.274	np2213	5.29	15.2	np2268	np2025	28.021-90.278
		*q08-7.1*	07	2020	29.356	np3234	5.79	16.5	np3306	np3260	11.799-38.372
		*q08-9.1*	09	2020	102.706	np4089	3.63	10.7	np4027	np4089	92.877-102.706
		*q08-11.1*	11	2020	25.150	lm2235	4.34	12.6	np4705	np4743	7.165-48.009
		*q08-12.1*	12	2020	91.578	lm2505	3.84	11.3	np5128	lm2505	90.018-91.578
9	*α*-Cubebene	*q09-8.1*	08	2020	43.255	np3484	3.29	9.8	np3484	np3488	43.255-46.045
10	*δ*-Cadinene	*q10-5.1*	05	2021	68.169	np2151	5.40	15.5	np2173	np1996	61.918-93.902
		*q10-8.1*	08	2020	46.045	np3488	3.93	11.5	np3484	np3572	43.255-50.123
		*q10-13.1*	13	2020	58.490	np5431	3.27	9.7	np5420	np5431	58.134-58.490
11	Calamenene	*q11-5.1*	05	2021	68.220	lm913	4.60	13.3	np2234	np1996	48.542-93.902
		*q11-6.1*	06	2021	104.015	np2736	3.84	11.2	np2736	np2749	104.015-111.134
		*q11-8.1*	08	2020	46.045	np3488	6.42	18.1	np3466	np3490	30.858-61.720
		*q11-13.1*	13	2020	58.134	np5420	4.06	11.9	np5416	np5432	57.538-59.298
12	(*E*)-Nerolidol	*q12-5.1*	05	2021	68.169	np2151	4.09	12.0	np2128	lm931	58.113-95.796
		*q12-11.1*	11	2020	32.715	np4770	3.08	9.1	lm2246	np4770	32.524-32.715
		*q12-11.2*	11	2020	49.003	np4801	3.19	9.5	lm2249	np4801	48.330-49.003
		*q12-12.1*	12	2021	92.460	hk1827	3.09	9.2	np5127	hk1826	90.002-92.998

Forty-two QTLs controlling monoterpene contents were identified with an average PVE of 12.6%. The LOD score of QTL *q01-3.1* associated with (*E*)-*β*-ocimene was the highest, which explained 18.5% of the phenotypic variation in 2020, and it was located between lm192 and np190 in LG03. Two stable QTLs associated (*E*)-*β*-ocimene were found to be located on LG05 and LG12. QTL *q01-5.1* was located between np2242 and np2002. It explained 15.40% of the phenotypic variation on average. Another QTL, *q01-12.1*, explained 12.9% and 10.5% of the phenotypic variation for 2020 and 2021 respectively, and the LOD peak was located at the position of 90.018 and 91.578. Two stable QTLs controlling linalool were identified on LG06 and LG12. QTL *q04-6.1* was located between markers np2749 and np2764, with an average PVE of 11.2% over two years. Another QTL, *q04-12.1*, was located on LG12, nearby the marker np5118 in 2020 and hk1788 in 2021, with the PVE of 10.0% and 13.1% respectively. The stable QTL, q07-5.1, associated with (*Z*)-nerol was detected on LG05. The LOD peak was present at the position of 55.485 cM in 2020 and 68.175 cM in 2021. The stable QTL associated with geraniol, q*08-5.2*, was identified on LG05. The confidence interval ranged from 28.021 to 90.278 cM over two years, and the overlapped region was from 38.893 to 89.868 cM, with a length of 50.975 cM. The QTL explained 14.8% and 15.2% of the phenotypic variance for 2020 and 2021 respectively.

For sesquiterpenes, 12 QTLs were identified on six linkage groups with an average PVE of 11.7%. The LOD score of QTL *q11-8.1* associated with calamenene was the highest, which explained 18.1% of the phenotypic variation in 2020. It was located between markers n3466 and np3490, in which *q09-8.1*, *q10-8.1*, and *q11-8.1* showed co-localization. Three QTLs (*q10-5.1*, *q11-5.1*, and *q12-5.1*) associated with *δ*-cadinene, calamenene, and (*E*)-nerolidol were located on LG05 between markers np2234 and lm931 in 2021, ranging from 48.542 to 95.796 cM. However, there were no stable QTL associated sesquiterpenes in the two years.

To summarize, two QTL clusters that carried more than 10 QTLs were found on LG05 and LG11, associated with both monoterpenes and sesquiterpenes (**Table 4**). The Chr11 cluster included 9 QTLs for monoterpenes and 2 QTLs for sesquiterpenes, ranging from 5.975 to 55.439 cM. The Chr05 cluster carried 3 QTLs for sesquiterpenes and 8 QTLs for monoterpenes, including 3 stable QTLs for (*E*)-*β*-ocimene, (*Z*)-nerol, and geraniol. It ranged from 18.227 to 103.417 cM, and the overlapped region for three stable QTLs was from 28.021 to 88.853 cM, with a length of 60.832 cM. It is worth noting that this cluster was associated with almost all aroma traits except linalool and *α*-cubebene, indicating that it might be key synthetic genes or potential regulatory network for volatile terpenes.

**Table 4:** QTL Clusters for volatile terpene contents in the ‘HD’ × ‘JX’ F_1_ population.

Cluster	Flanking markers	Trait	QTL
Chr05 cluster	np2767-lm942	(*E*)-*β*-Ocimene	*q01-5.1^#^*
		*cis*-Linalool oxide (furanoid)	*q02-5.1*
		*cis*-Linalool oxide (furanoid)	*q02-5.2*
		*trans*-Linalool oxide (furanoid)	*q03-5.1*
		*trans*-Linalool oxide (pyranoid)	*q05-5.1*
		*α*-Terpineol	*q06-5.1*
		(*Z*)-Nerol	*q07-5.1^#^*
		Geraniol	*q08-5.1*
		Geraniol	*q08-5.2^#^*
		*δ*-Cadinene	*q10-5.1*
		Calamenene	*q11-5.1*
		(*E*)-Nerolidol	*q12-5.1*
Chr11 cluster	np4616-np4837	(*E*)-*β*-Ocimene	*q01-11.1*
		*cis*-Linalool oxide (furanoid)	*q02-11.1*
		*cis*-Linalool oxide (furanoid)	*q02-11.2*
		*cis*-Linalool oxide (furanoid)	*q02-11.3*
		*trans*-Linalool oxide (furanoid)	*q03-11.1*
		*trans*-Linalool oxide (furanoid)	*q03-11.2*
		*α*-Terpineol	*q06-11.1*
		(*Z*)-Nerol	*q07-11.1*
		Geraniol	*q08-11.1*
		(*E*)-Nerolidol	*q12-11.1*
		(*E)*-Nerolidol	*q12-11.2*

^#^Indicated stable QTL over two years.

### 3.5 Gene expression profiling in Chr05 QTL cluster based on RNA-seq analysis

**Figure 4:**
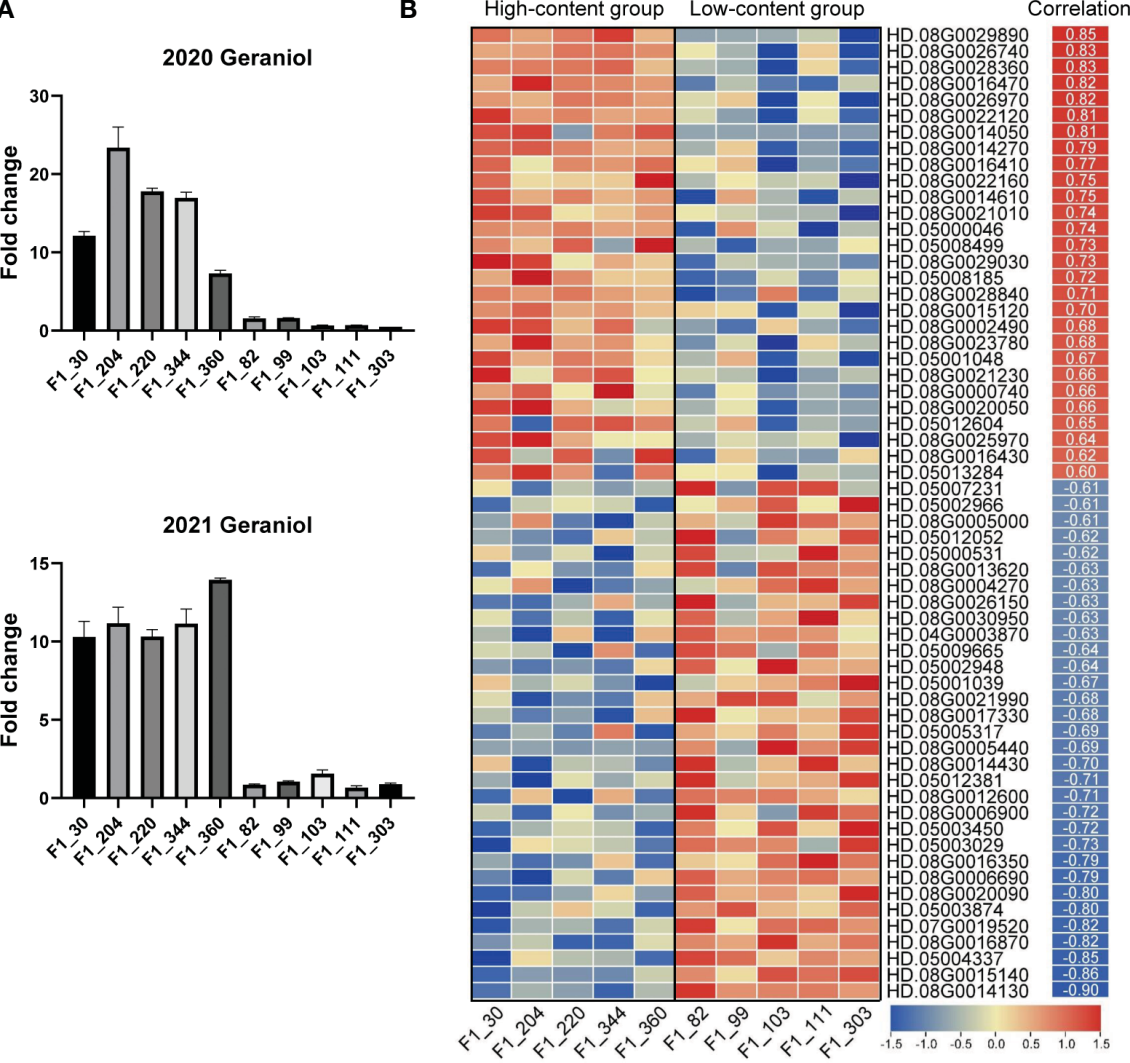
RNA-Seq data analysis of candidate genes for volatile terpene contents in tea plant. The contents of geraniol in ten selected F_1_ individuals in 2020 and 2021 **(A)**. Results are expressed as mean ± SEM (*n* = 3). Expression patterns of candidate genes in Chr05 cluster **(B)**. Pearson’s correlations between geraniol contents and the expression levels of candidate genes in 2021 are shown on the right of heatmap.

Transcriptomic analysis is a powerful tool for screening candidate genes in F_1_ hybrid progeny (Huang et al., 2022a). To further investigate the candidate genes in Chr05 cluster, we conducted comparative transcriptomics analyses on ten F_1_ individuals with distinct contents of volatile terpenes in 2021, including five high-content individuals and five low-content individuals. According to the VIP values and the PVE of QTLs mentioned above, geraniol was selected as representative volatile terpene. The average fold changes of geraniol content in the two groups were more than ten times in the two years (**Figure 4A**). Overall, ~1.38 billion paired-end reads were obtained by Illumina sequencing, with an average of 44.8 million reads per library. The average Q20 and Q30 were 96.91% and 91.73% respectively. Approximately 89.22% of the reads were mapped against the 'HD' genome, with on average 85.39% of uniquely mapped reads. The correlations between gene expression levels and geraniol contents in 2021 were carried out. After filtering for low correlation genes (Pearson’s correlation |*r*| < 0.6), a comparison of differentially expressed genes (DEGs) (Average FPKM of higher group > 5, Padj < 0.05 and |fold change| > 1.5) among different groups was illustrated in the heatmap (**Figure 4B**). In comparison to the low-content group, 60 (28 up-regulated and 32 down-regulated) genes were found to be differentially expressed in the Chr05 cluster. Among them, an aldehyde dehydrogenase gene (HD.08G0014050) showed the most significant up-regulated, while the most down-regulation was observed at more than 14 folds for transcript HD.08G0005440 encoding ankyrin repeat-containing protein. The information on DEGs was shown in **Table S2**.

Among the up-regulated genes in the Chr06 cluster, four genes directly related to terpene synthesis were found, including three terpene synthase (TPS) genes (HD.08G0016410, HD.08G0016430, and HD.08G0016470) and a 1-deoxy-D-xylulose-5-phosphate synthase (DXS) gene (HD.08G0002490). HD.08G0016410 has the highest average expression level among the three *TPS* genes, the average FPKM in the high-content group was 49. The max fold change was observed in HD.08G0016470 (fold change = 5.27). The correlation coefficients with geraniol contents were 0.77, 0.62, 0.82 and 0.68 for HD.08G0016410, HD.08G0016430, HD.08G0016470, and HD.08G0002490, respectively. These four genes could be the candidate synthase genes for volatile terpene accumulation.

Transcription factors (TFs) are master regulators that orchestrate the regulation of many different aspects of plant development and responses (Shen et al., 2022; Zhao et al., 2022). In order to find potential regulatory genes of volatile terpene contents, we also searched for transcription factors in DEGs. As a result, a GRAS family gene (HD.08G0021230) showed significant up-regulation in the high-content group (fold change = 2.03). In addition, a strong positive correlation (*r* = 0.88) was found between geraniol content and the expression levels of a *bHLH* gene (HD.08G0006200).

**Figure 5:**
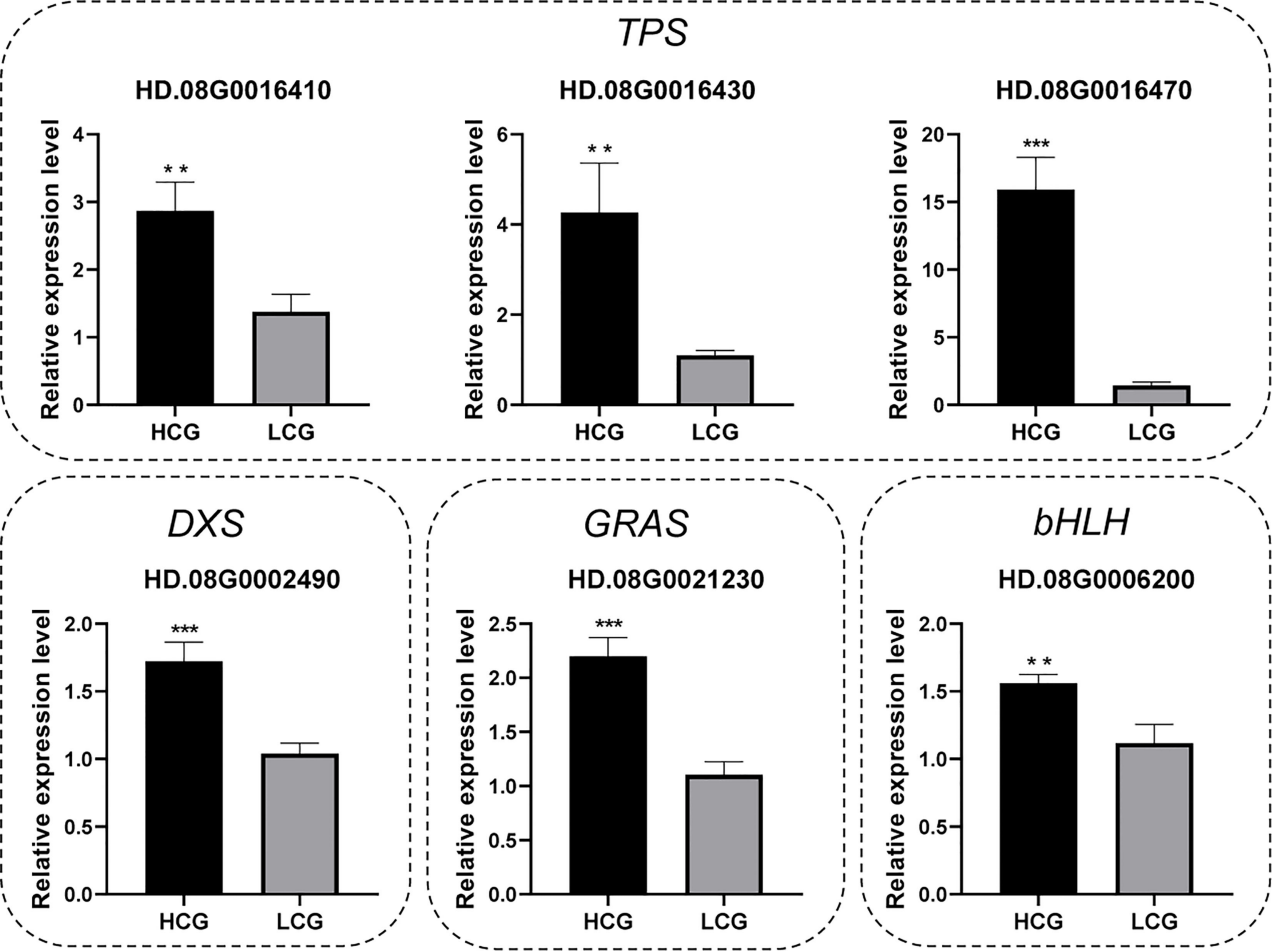
Verification of the candidate gene expression levels by qRT–PCR. Results are expressed as mean ± SEM (*n* = 15, ∗∗∗*P* < 0.001, ∗∗*P* < 0.01). HCG: high-content group, LCG: low-content group.

To verify the accuracy of RNA-Seq results, four candidate genes related to terpene synthesis pathway and two TFs were selected for for qRT-PCR analysis. As shown in **Figure 5**, all candidate genes showed highly significant differences (*n* = 15, *P* < 0.01) between high-content group and low-content group, consist with RNA-Seq results.

## 4 Discussion

### 4.1 QTL identification for volatile terpene contents

Mapping QTLs of aroma traits is challenging. Volatiles are influenced by a variety of environmental factors, and the determination of aroma is more complicated than soluble substances such as amino acids and caffeine. QTL mapping of fruit aroma compounds has been studied in several plants. For instance, aroma volatiles of *Citrus reticulata* were investigated in the 116 ‘Fortune’ × ‘Murcott’ F_1_ progeny using GC–MS, and a total of 206 QTLs were identified for 94 volatile compounds (Yu et al., 2017). In cucumber, a total of 28 QTLs associated with C6 and C9 aldehydes and alcohols were screened in a RIL population developed from Q16 × Q24 (Sun et al., 2022). Similar studies have been done on kiwifruit and apple (Yauk et al., 2015; Zeng et al., 2020). Nevertheless, QTL mapping of aroma phenotypes in tea plant has not yet been reported.

A fitting population and high-density genetic map suggested an effective way to identify QTLs related to aroma phenotypes. In this study, we constructed a high-density SNP linkage map using 148 F_1_ individuals developed from the controlled cross between two high-aroma oolong tea cultivars 'HD' and 'JX' using GBS. Compared with previously reported linkage maps, we achieved a higher map density as well as shorter marker distances (0.47 cM), which is comparable to the genetic map derived from the cross of ‘Longjing 43’ and ‘Baihaozao’ with an interval distance of 0.40 cM (Xu et al., 2018a), higher than that from the crossed between ‘Longjing 43’ and ‘Baijiguan’ (0.69 cM) (Huang et al., 2022b). Briefly, we identified 54 QTLs associated with 12 volatile terpenes for the first time. Previous studies have demonstrated the contribution of these targeted aromas to tea products, especially for those compounds with high contents and low thresholds such as geraniol, linalool and linalool oxides (Schuh and Schieberle, 2006; Xiao et al., 2022). Among them, six stable QTL were validated in these two years, the PVE of two stable QTLs (*q01-5.1* and *q08-5.2*) for (*E*)-*β*-ocimene and geraniol were higher than 15%, which could be considered as major QTLs. Further, we found more than ten QTLs clustered in the same region on LG05 and LG11. The same result was also found in grape, major QTLs located at the top LG5 found for 14 monoterpenes, explained 14.7–48.1% of the total phenotypic variance (Koyama et al., 2022). These results indicated the presence of the same synthetic or regulatory pathway for those volatile terpenes in this study.

### 4.2 Candidate genes related to volatile terpene contents in Chr05 QTL cluster

Terpenoids as the most abundant class of natural products have been well-studied in plants (Singh and Sharma, 2014). In higher plants, terpenoids are synthesized either *via* the methylerythritol phosphate (MEP) pathway or the mevalonate (MVA) pathway (Dudareva et al., 2006). The common precursors for terpenoid synthesis, dimethylallyl pyrophosphate (DMAPP) and isopentenyl pyrophosphate (IPP), are produced independently by the MVA and MEP pathways, and it has been shown that there might be crosstalk and competition for substrates between the two pathways (Wu et al., 2006). The precursors of mono-, sesqui- and diterpenes, including geranylgeranyl pyrophosphate (GPP), farnesyl pyrophosphate (FPP) and geranylgeranyl pyrophosphate (GGPP), are formed by the condensation of 1:1, 2:1, and 3:1 IPP and DMAPP respectively, followed by TPS to form various terpenoids (Chen et al., 2011). Among the synthetic genes of terpenoid backbone, *DXS* is the first rate-limiting enzyme in the MEP pathway (Tian et al., 2022). The enzymatic activity of DXS was significantly correlated with the content of monoterpenes in grape (Dalla Costa et al., 2018). In this study, a differentially expressed *DXS* gene (HD.08G0002490) was found in Chr05 cluster. The expression levels of it showed positively correlated with the contents of geraniol. As we mentioned above, the Chr05 cluster has associated with almost all aroma traits and most of the volatile terpene contents showed a positive correlation. Hence, the substantial differences in expression level of this gene may suggest its essential role for total volatile terpene accumulation.

Most monoterpenes and sesquiterpenes synthesized by TPSs using GPP and FPP as direct substrates, respectively (Bohlmann et al., 1998). In tea plant, some TPS genes involved in the synthesis of (*E)*-nerolidol, *α*-farnesene, and linalool have been reported (Zhou et al., 2017; Liu et al., 2018; Wang et al., 2019a). But the mechanism of synthesis of some other volatile terpenes such as geraniol and nerol have remained unclear. Here, we found nine TPS genes in the Chr05 cluster according to gene annotation and three genes showed up-regulation in the high-content group which were consistent with the phenotypes. To predict the function of these three genes, we constructed a neighbor-joining phylogenetic tree that contained representative TPS sequences from several plant species (**Figure S2**). The results showed that all three genes are clustered into the TPS-b subclade, which belongs to the monoterpene synthase subfamily (Bohlmann et al., 1998). Previous studies have shown that a single terpene synthase may produce multiple products of terpenoid. For instance, a TPS-b *α*-farnesene synthase from apple (MdAFS1), can also synthesize the (*E*)-*β*-ocimene (Green et al., 2011). Likewise, distinct transcript splicing regulation of terpene synthase gene *CsLIS/NES* leads to the transformation of enzymatic function (nerolidol synthase or linalool synthase) in tea plant (Liu et al., 2018). Therefore, their specific functions merits further investigations.

Several TFs have been found to activate or repress terpenoid metabolism by binding to the promoter regions of TPSs or genes coding. LcERF19, an AP2/ERF transcription factor, could binding to *LcTPS42* promoter and promoted its activity, which is related to the synthesis of geranial and neral in *Litsea cubeba* (Wang et al., 2022a). Belonging to the bHLH family, MYC2 has been shown to be involved in JA-regulated volatile aroma synthesis under stress in tea plant (Li et al., 2019). We found several TFs in Chr05 QTL cluster, such as bHLH, MYB, GRAS, and WRKY. Among them, the expression levels of a *GRAS* gene and a *bHLH* gene showed significant differences. In addition to bHlH family, it has been shown that the GRAS family is also involved in the regulation of volatile terpenes. For example, a scarecrow‐like (SCL) subfamily transcription factor SlSCL3 led to a significant decrease of expression of the corresponding TPS genes and volatile terpene contents when its transcription level was down-regulated in tomato (Yang et al., 2021). The results of QTL identification together with differences in expression suggested the potential roles of these two candidate TFs for volatile terpene accumulation. Further study on determining whether they can directly bind to the candidate *TPS* genes is warranted.

## 5 Conclusion

In summary, a high-density genetic linkage map of tea plant was constructed using GBS. The linkage map contained 15 linkage groups with a low inter-marker distance of 0.47 cM. A total of 42 QTLs associated with monoterpene contents and 12 QTLs associated with sesquiterpenes contents were identified. The high-density linkage map will serve as a foundation for tea genetic improvement. In the future, we will continue to adopt a strategy to fine map and identify QTL mapping intervals to further identify favorable target genes and verify their function.

## Data availability statement

The datasets presented in this study can be found in online repositories. The names of the repository/repositories and accession number(s) can be found at https://www.ncbi.nlm.nih.gov/bioproject/PRJNA916459/.

## Author contributions

LC and JM conceived and designed the experiments. SC, XL, and YL performed the experiments. SC, XL, and JC analyzed the data. SC, JM, and LC wrote the manuscript. All authors contributed to the article and approved the submitted version.
