# Supplementary material for: Identification of QTL controlling volatile terpene contents in tea plant (Camellia sinensis) using a high-aroma ‘Huangdan’ x ‘Jinxuan’ F1 population
Source: Front Plant Sci. 2023 Mar 29;14:1130582. doi: 10.3389/fpls.2023.1130582 (PMC10090551; doi:10.3389/fpls.2023.1130582)
Supplement: Supplementary file 1 [file DataSheet_1.docx]

Supplementary Material

Identification of QTL controlling volatile terpene contents in tea plant (*Camellia sinensis*) using a high-aroma ‘Huangdan’ x ‘Jinxuan’ F_1_ population

**Si Chen, Xuanye Li, Yujie Liu, Jiedan Chen, Jianqiang Ma*, Liang Chen***

**Correspondence:** Dr. Jianqiang Ma, majianqiang@tricaas.com; Dr. Liang Chen, liangchen@tricaas.com

## Supplementary Table

**Supplementary Table S1.** The information on 3770 markers used in the linkage map.

**Supplementary Table S2.** The information on differentially expressed genes in the Chr05 cluster.

**Supplementary Table S3.** Primers for qRT-PCR.

## Supplementary Figures

**Supplementary Figure S1.** Segregation types of polymorphic SNP markers.

**Supplementary Figure S2.** Phylogenetic analysis of Candidates *TPS* genes. The neighbor-joining phylogenetic tree was generated by MEGA 7 software. Bootstrap values from 1000 replicates were used to assess the robustness of the tree. Candidates *TPS* genes sequence from the current study is highlighted in red box.
